# Identification of a candidate gene for a QTL for spikelet number per spike on wheat chromosome arm 7AL by high-resolution genetic mapping

**DOI:** 10.1007/s00122-019-03382-5

**Published:** 2019-06-28

**Authors:** Saarah Kuzay, Yunfeng Xu, Junli Zhang, Andrew Katz, Stephen Pearce, Zhenqi Su, Max Fraser, James A. Anderson, Gina Brown-Guedira, Noah DeWitt, Amanda Peters Haugrud, Justin D. Faris, Eduard Akhunov, Guihua Bai, Jorge Dubcovsky

**Affiliations:** 1 Department of Plant Sciences, University of California, Davis, CA 95616 USA; 2 Department of Agronomy, Kansas State University, Manhattan, KS 66506 USA; 3 Department of Soil and Crop Sciences, Colorado State University, Fort Collins, CO 80523 USA; 4 Department of Agronomy and Plant Genetics, University of Minnesota, St. Paul, MN 55108 USA; 5 USDA-ARS Plant Science Research, Raleigh, NC 27695 USA; 6 Department of Crop and Soil Sciences, North Carolina State University, Raleigh, NC 27695 USA; 7 Department of Plant Sciences, North Dakota State University, Fargo, ND 58102 USA; 8 USDA-Agricultural Research Service, Cereal Crops Research Unit, Edward T. Schafer Agricultural Research Center, Fargo, ND 58102 USA; 9 Department of Plant Pathology, Kansas State University, Manhattan, KS 66506 USA; 10 USDA-ARS, Hard Winter Wheat Genetics Research Unit, Manhattan, KS 66506 USA; 11 Howard Hughes Medical Institute, Chevy Chase, MD 20815 USA

## Abstract

**Key message:**

A high-resolution genetic map combined with haplotype analyses identified a wheat ortholog of rice gene *APO1* as the best candidate gene for a 7AL locus affecting spikelet number per spike.

**Abstract:**

A better understanding of the genes controlling differences in wheat grain yield components can accelerate the improvements required to satisfy future food demands. In this study, we identified a promising candidate gene underlying a quantitative trait locus (QTL) on wheat chromosome arm 7AL regulating spikelet number per spike (SNS). We used large heterogeneous inbred families ( > 10,000 plants) from two crosses to map the 7AL QTL to an 87-kb region (674,019,191–674,106,327 bp, RefSeq v1.0) containing two complete and two partial genes. In this region, we found three major haplotypes that were designated as H1, H2 and H3. The H2 haplotype contributed the high-SNS allele in both H1 × H2 and H2 × H3 segregating populations. The ancestral H3 haplotype is frequent in wild emmer (48%) but rare (~ 1%) in cultivated wheats. By contrast, the H1 and H2 haplotypes became predominant in modern cultivated durum and common wheat, respectively. Among the four candidate genes, only *TraesCS7A02G481600* showed a non-synonymous polymorphism that differentiated H2 from the other two haplotypes. This gene, designated here as *WHEAT ORTHOLOG OF APO1* (*WAPO1*), is an ortholog of the rice gene *ABERRANT PANICLE ORGANIZATION 1* (*APO1*), which affects spikelet number. Taken together, the high-resolution genetic map, the association between polymorphisms in the different mapping populations with differences in SNS, and the known role of orthologous genes in other grass species suggest that *WAPO-A1* is the most likely candidate gene for the 7AL SNS QTL among the four genes identified in the candidate gene region.

**Electronic supplementary material:**

The online version of this article (10.1007/s00122-019-03382-5) contains supplementary material, which is available to authorized users.

## Introduction

Common wheat (*Triticum aestivum* L.) and durum wheat (*T. turgidum* subsp. *durum* (Desf.) Husn.) provide more than 20% of the calories and protein consumed by the human population (FAOSTAT 2017). Faster increases in wheat grain yield are required to meet the demands of a growing human population, but progress in our understanding of the genes and gene networks that control grain yield has been hampered by the low heritability of this trait. A strategy to address this problem is to divide total grain yield into yield components such as spikes per unit of area, grain number per spike (GNS) and average grain weight, which usually exhibit higher heritability (Zhang et al. 2018). GNS can be further divided into grain number per spikelet and spikelet number per spike (SNS), which is the focus of this study.

SNS is determined by the number of lateral spikelet meristems generated by the spike meristem before it transitions to a terminal spikelet. This number is determined at an early stage of wheat reproductive development and is less affected by growing conditions than other yield components (Zhang et al. 2018). By contrast, GNS is affected by abortion of developing florets and grains during a longer portion of the growing season (Gonzalez et al. 2011; Zhang et al. 2018). The earlier developmental determination of SNS may explain its higher heritability relative to GNS. In a recent genome-wide association study (GWAS) including 262 common wheat photoperiod-insensitive spring lines, heritability values for SNS (average *H*^2^ = 0.84) were ~ 30% higher than those for GNS (average *H*^2^ = 0.64) (Zhang et al. 2018).

The high heritability of SNS facilitated the identification of a highly significant and consistent association (*P* < 0.0001) between this trait and SNP marker IWA5912 on chromosome arm 7AL in the five field trials where the spring wheat panel was evaluated (Zhang et al. 2018). Strong associations between markers in this region and GNS or SNS were also reported in other GWAS, including 329 soft red winter wheats in the USA (Ward et al. 2019), 209 winter wheats in Germany (Boeven et al. 2016; Wurschum et al. 2018) and 220 common winter wheat European varieties registered between 1966 and 2013 (Voss-Fels et al. 2019).

This region on wheat chromosome arm 7AL has been also associated with differences in SNS and GNS in biparental populations in both hexaploid wheat and tetraploid wheat. The IWA5912 peak of the SNS QTL identified in the GWAS was validated in the biparental population ‘Berkut’ × ‘RAC875’ in four separate experiments (Zhang et al. 2018). The same chromosome region has been also associated with QTLs for SNS, GNS and/or grain yield in crosses between ‘Chinese Spring’ and breeding line ‘SQ1’ (Quarrie et al. 2006), the Tibetan semi-wild wheat ‘Q1028’ and the Chinese cultivar ‘Zhengmai 9023' (Luo et al. 2016), and between other Chinese wheat cultivars (Xu et al. 2014; Zhai et al. 2016). In tetraploid wheat, the IWA5912 marker was also found at the peak of a strong QTL for SNS in a cross between durum wheat and cultivated emmer (*T. turgidum* subsp. *dicoccon* (Schrank ex Schuebl.) Thell.) (Faris et al. 2014).

In addition, QTLs for grain weight have been mapped on the same chromosome region as the 7AL SNS QTL. Increases in grain number are frequently associated with decreases in average grain weight, particularly in plants that are ‘source’ limited (Faris et al. 2014). Therefore, co-located QTLs of opposite effects for GNS and grain weight can represent pleiotropic effects of the same gene. This seems to be the case for a strong kernel weight QTL identified on a 2.2-Mb region of chromosome arm 7AL (including IWA5912) in a cross between a Chinese facultative hard wheat and a USA soft winter wheat (Su et al. 2016). Similarly, the kernel weight QTL (peak marker only 1 Mb from IWA5912) reported in a cross between tetraploid and hexaploid wheat (Kalous et al. 2015) may also be a pleiotropic effect of a gene affecting GNS.

These studies defined a yield components QTL region between 672.0 and 674.3 Mb in the 7A pseudomolecule of the Chinese Spring genome released by the International Wheat Genome Sequencing Consortium (IWGSC), henceforth RefSeq v1.0 (IWGSC 2018). This region includes 61 genes (27 high confidence and 34 low confidence) (Keeble-Gagnere et al. 2018), which segregates in multiple breeding programs. In this study, we precisely mapped the locus affecting SNS to an 87-kb region containing only two complete genes and two partial genes. Among those genes, only one showed a non-synonymous polymorphism that co-segregated with differences in SNS in different mapping populations. This wheat gene is orthologous to *ABERRANT PANICLE ORGANIZATION 1* (*APO1*), a gene known to affect panicle development and spikelet number in rice (Ikeda et al. 2007). Based on the genetic data, expression profile and predicted function, we suggest that the wheat ortholog of *APO1* is the best candidate gene for the SNS QTL among the four genes present in the candidate region.

## Materials and methods

### Plant materials

Two high-resolution maps were constructed using heterogeneous inbred families (HIFs) derived from heterozygous recombinant inbred lines (RILs) identified in the crosses ‘Berkut’ ×‘RAC875’ (Zhang et al. 2018) and ‘Ning7840’ ×‘Clark’. The segregating populations generated from these crosses were previously shown to segregate for the SNS QTL on chromosome arm 7AL. Berkut (Irene/Babax//Pastor) is a spring wheat cultivar developed by CIMMYT, and RAC875 (RAC655/3/Sr21/4*Lance//4*Bayonet) is a spring wheat breeding line from South Australia. Ning7840 (Aurora/Anhui11//Sumai3) is a hard red facultative wheat line from China, and Clark (Beau//65256A1-8–1/67137B5-16/4/Sullivan/3/Beau//5517B8-5–3-3/Logan) is a soft red winter wheat cultivar from Purdue University, IN, USA.

The effect of the candidate gene region was validated in one additional tetraploid and three hexaploid biparental mapping populations. In tetraploid wheat, we used an F_7:8_ RIL population including 197 lines from the cross between the durum wheat cultivar ‘Ben’ (PI 596557) and cultivated emmer accession PI 41025. The RILs were evaluated in two field experiments in Prosper, North Dakota, in 2017 and 2018 planted in a randomized complete block design (RCBD) with three replications (eight spikes measured per replication).

In hexaploid wheat, the effect of the candidate gene region was validated in three biparental populations. The first one included 327 F_6:7_ RILs from the cross between soft winter wheat cultivars ‘LA95135’ (LA90239A29/LA8644A3-3–2-P2) and ‘SS-MPV57’ (FFR555W/3/Lovrin 29/Tyler//Redcoat*2/Gaines, henceforth MPV57), which were evaluated for SNS in two field experiments in Raleigh and Kinston (NC, USA). The experiments were planted as augmented designs with two replications, and at least six spikes were measured per replication. The second hexaploid population included 223 F_5:6_ RILs from the cross between hard white winter wheats ‘CO940610’ (KS87H22/MW09) and ‘Platte’ (Tesia 79/Chat 'S'//Abilene). Two replications of the complete RIL population were grown as single rows in Fort Collins, CO, under irrigation. From each row, 10 spikes were randomly selected and measured to determine the average SNS. The last hexaploid population was derived from the cross between hard spring wheat lines ‘MN98550-5’ (BacUp/McVey) and ‘MN99394-1’ (SD3236/SBF0402). This population included 138 F_6:9_ RILs that were evaluated as single rows at the University of Minnesota (Crookston, MN, USA) in 2017. Ten spikes per row were evaluated for SNS and averaged for further data analysis.

Exome-capture data were obtained for four durum wheats, one *T. turgidum* subsp. *dicoccoides* (Koern. ex Asch. & Graebn.) Thell. (henceforth wild emmer) and 49 hexaploid wheats including parental lines of the WheatCAP project (https://www.triticeaecap.org/wheatcap-germplasm-list/) using an assay previously described (Krasileva et al. 2017). Sequences for the candidate gene were also obtained from the published genome of *T. turgidum* subsp. *dicoccoides* Zavitan (Avni et al. 2017) and from 14 genomes sequenced in the Wheat Pan Genome project (https://www.10wheatgenomes.com/) generously provided by Dr. Curtis Pozniak. The candidate gene region was also sequenced for the parental lines of the different mapping populations presented in this study. These sequences were deposited in GenBank under the following accession numbers: Berkut (MK463869), RAC875 (MK463870), Rusty (MK463871), LA95135 (MK463872), MPV57 (MK463873), MN99394-1 (MK463874), MN98550-5 (MK463875), Clark (MK463876), Ben (MK463877), CO940610 (MK463878), Ning7840 (MK463879), PI 41025 (MK463880), and Platte (MK463881).

Markers developed for diagnostic polymorphisms detected in the candidate gene were used to evaluate 74 diploid *T. urartu*, 21 wild emmer, 71 cultivated emmer, 364 durum and 897 common wheat accessions. The common wheat accessions include two panels genotyped in previous studies. The first one includes photoperiod-insensitive spring wheat cultivars mainly from North America and CIMMYT (Zhang et al. 2018). This panel was genotyped with the Illumina Infinium iSelect 90-K wheat SNP array (henceforth, 90-K SNP array. Illumina Inc., San Diego, CA, USA) (Wang et al. 2014). The second panel includes a worldwide collection of diverse landraces, cultivars (modern and old), and different *T. aestivum* subspecies (He et al. 2019). This panel excluded cultivars with extensive shared ancestry to maximize the probability of including unique genotypes. These accessions were genotyped with a wheat exome-capture assay that targeted 107 Mb of non-redundant low-copy regions in the wheat genome (Jordan et al. 2015). This exome-capture assay is different from the one developed by Krasileva et al. (2017), which was focused exclusively in coding regions (referred hereafter as wheat exome-capture v2). Genome coordinates presented in this study are based on RefSeq v1.0 assembly and gene annotations on the updated RefSeq v1.1 annotation (IWGSC 2018).

### Map construction and QTL mapping

The 75 RILs of the Berkut × RAC875 population (henceforth B×R) were genotyped with the 90-K SNP array (Wang et al. 2014). The 7518 polymorphic SNPs detected in this assay were used to construct a genetic map using the R package ASMap (Taylor and Butler 2017). For the initial map, only those markers that were not co-located with other markers and had < 10% missing values were included. The function ‘mstmap’ was used to group and order markers with *P* < 1e^−6^ and calculate genetic distances using the ‘kosambi’ function. We used the position of the SNPs in RefSeq v1.0, to merge separate linkage groups that originated from the same chromosome. Markers with < 20% missing values were added as the last step.

QTL mapping for SNS, kernel weight and grain yield in the B×R population was performed using R/qtl package (Broman et al. 2003). QTLs were identified by simple interval mapping using the function ‘scanone’ with the extended Haley–Knott method (Feenstra et al. 2006). A LOD 2.0 threshold was used to identify significant QTLs. Later, the multiple QTL mapping (MQM) method, as implemented in R/qtl, was used to determine significant QTLs, find QTL peak positions and calculate the percent of variation explained by each QTL. Phenotypic data were obtained from previous field experiments at the UC Experimental Field Station in Davis, CA, and the UC Desert Research and Extension Center in El Centro, CA (Zhang et al. 2018). In each location, plants were grown under both full irrigation and terminal drought (no irrigation after booting stage) (Zhang et al. 2018). Field variation was adjusted using the mixed linear model in the R package ‘lme4’ (R Core Team 2017; Bates et al. 2015). ANOVAs were performed separately for each of the four treatments and also in a combined analysis using treatments as blocks. The best linear unbiased predictors (BLUPs) of traits across environments were obtained for later analysis by fitting a linear mixed model, where both genotypes and environments were treated as random effects.

### Construction of the high-resolution maps

Two high-resolution maps were constructed independently at the University of California Davis (UCD) and USDA Small Grains Genotyping Lab at Kansas State University (USDA). Only in the final stage, when we realized that we were converging on the same region, we decided to combine the results in a single publication. The UCD map was based on the B×R populations described above and was constructed in two phases. In the first phase, we screened 617 F_2_ progeny derived from two independent F_5_ and F_6_ HIFs with markers IWB713 (670,767,495 bp) and IWB53096 (679,896,953 bp). Plants showing recombination events within the target region were self-pollinated, and homozygous recombinant and non-recombinant sister lines were selected and evaluated for SNS in replicated greenhouse and field experiments. For the second phase of the high-resolution mapping, we screened 1208 segregating F_6:3_ plants with the new markers flanking the smaller region for additional recombinant lines. Progeny from lines carrying the four closest recombination events were phenotyped for SNS in two independent greenhouse experiments (23–34 plants per progeny test) and were further validated in a field experiment using 10 replications per line. Greenhouse temperatures varied between 22.8 ± 1.6 °C during the day and 20.3 ± 0.3 °C during the night, and natural light was supplemented for 14 h with artificial light.

The USDA high-resolution map of the Ning7840 × Clark population (designated N×C hereafter) was derived from six F_4_ HIFs. These HIFs were identified in a secondary RIL population developed from the cross between N×C RILs L115 and L118 that differed in the 7AL QTL haplotypes. We first genotyped 4219 F_4:5_ plants using flanking Kompetitive Allele Specific PCR (KASP) markers IWB7435 (671,218,901 bp) and IWA5167 (679,955,879 bp), and identified 93 heterozygous recombinant plants within the 8.7-Mb QTL region. From the progenies of these recombinant lines, we conducted two additional screening cycles including 2277 and 3948 plants, respectively, to identify homozygous recombinant lines for phenotyping and to generate new recombination events in the region. Progeny of the lines showing critical recombination events were evaluated for SNS in replicated greenhouse experiments using 22–59 plants per progeny test. Greenhouse temperatures varied between 22 ± 5 °C during the day and 17 ± 3 °C during the night, and natural light was supplemented for 12 h with high-pressure sodium lights.

Lines from the two high-resolution mapping populations showing recombination events in the target region were genotyped with additional markers generated from polymorphisms identified using the wheat exome-capture v2, the 90-K SNP array and the 660-K SNP array (https://wheat.pw.usda.gov/ggpages/topics/Wheat660_SNP_array_developed_by_CAAS.pdf). KASP assays were developed to screen the recombinant lines and their progeny. The large number of replications in the progeny tests, the homogeneous genetic background of the HIFs, and the high heritability of the trait allowed us to map the SNS QTL as a simple Mendelian locus.

### Expression

We used qRT-PCR to compare the levels of expression of the genes within the candidate region in the basal, central and distal portions of the developing spikes at the floret primordia stage. The developing spikes were sectioned transversally in three equal parts designated as basal, central and apical. *WAPO-A1* transcripts were amplified using A-genome-specific primers UFO-A-RT-F2 (5′-CTCACTCACTCTCACTCCACG-3′) and UFO-A-RT-R2 (5′-GGTGGTGAGGCAGTAGGTTC-3′) that showed an efficiency of 92%.

The same primers were used to compare the transcript levels of the candidate gene in developing spikes at the floret primordia stage in near isogenic HIF lines carrying the Berkut and RAC875 alleles. We analyzed four replications for each genotype, each consisting of pooled developing spikes from 9 to 12 plants at the same developmental stage. Plants were grown under 14 h of light (22 °C, 330 mol intensity) and 10 h without light (17 °C) in a growth chamber.

The qRT-PCRs were performed on an ABI 7500 Fast Real-Time PCR System (Applied Biosystems) using Fast SYBR GREEN Master Mix. The PCR cycles included 2 min at 95 °C, followed by 40 cycles of 5 s at 95 °C and 30 s at 60 °C. Transcript levels were expressed as fold-*ACTIN* levels (the number of molecules in the target/the number of *ACTIN* molecules) using the 2^ΔCT^ method as described before (Pearce et al. 2013).

## Results

### QTL mapping

**Fig. 1:**
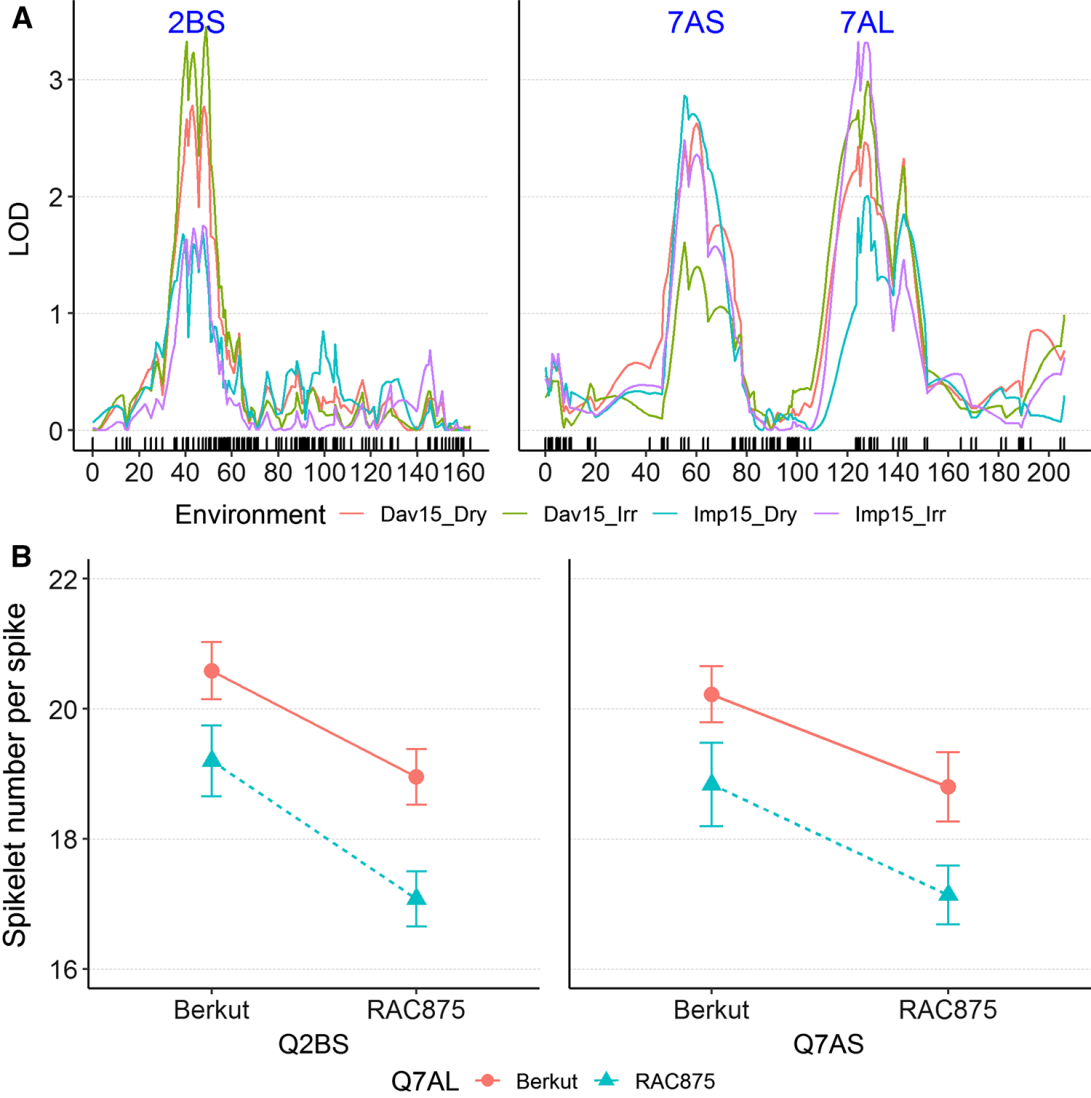
QTLs for SNS detected in the Berkut × RAC875 population. **a** Results from field experiments performed in Davis (Dav.) and Imperial Valley (Imp.) California, under both full irrigation (Irr.) and terminal drought (Dry). Values in the X-axis indicate position in the chromosome in centiMorgans starting from the distal end of the short arm. **b** Interaction graph between the SNS QTLs in 7AL and 2BS (left) and in 7AL and 7AS (right). Values are averages ± standard errors of the means. No significant interactions were detected among QTL, which is reflected in the parallel lines in the interaction graphs

The 7518 polymorphic SNPs detected between Berkut and RAC875 were used to construct a map with 987 unique loci (Supplementary File S1). Using SNS data previously collected from four field experiments (two California locations, both under full and restricted irrigation) (Zhang et al. 2018), we identified three significant QTLs for SNS on chromosome arms 2BS, 7AS and 7AL that were consistent across environments (Fig. 1a). Their peak markers, locations in the genetic map and in the wheat reference genome, and statistics (LOD score, additive effect and percent of explained variation) are described in Table S1. An ANOVA using the four environments as blocks and peak markers of the three SNS QTLs as factors showed highly significant effects for all three QTLs, but no significant interactions (Table S1 and Fig. 1b). This result indicated that HIFs fixed for different combinations of the 2BS and 7AS QTLs could be equally useful to map the 7AL QTL, which is the focus of this study.

### High-resolution map of the SNS 7AL QTL in the Berkut × RAC875 population

In the screening of the Berkut × RAC875 RILs, we found that RIL23 (F_5_) and RIL42 (F_6_) were heterozygous for flanking markers IWB53096 and IWB713 and selected them to generate HIF populations. Although HIF23 showed a higher SNS than HIF42, both showed significant differences in SNS between sister lines homozygous for the Berkut and RAC875 haplotypes (Fig. S1A). The pooled SNS standard deviations obtained in field experiment using RILs were approximately fourfold higher than those obtained using HIFs. The lower variability of the HIFs increased the statistical power and reduced the size of the progeny tests required to detect significant differences between the two haplotypes (Fig. S1B).

**Fig. 2:**
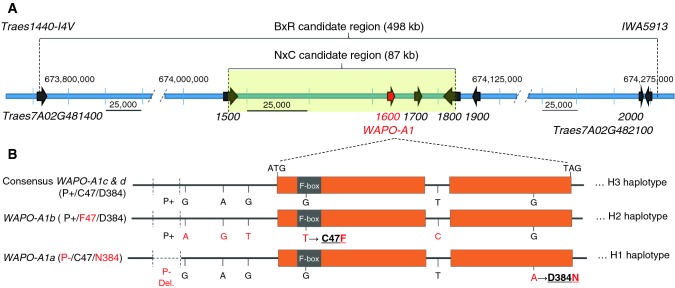
Physical map of the candidate gene region in chromosome arm 7AL and candidate genes. **a** Candidate gene region based on the B×R and N×C Arrows represent high-confidence genes (5′–3′ direction, RefSeq v1.0 coordinates and RefSeq v1.1 annotation). Note the different scales in the central region and distal regions. Complete gene names are provided only for the flanking genes, and only the last four numbers are provided for the other genes. Candidate gene *TraesCS7A02G481600* (= *WAPO-A1*) is highlighted in red. **b** Gene models of *WAPO-A1* main alleles. Alleles *WAPO-A1a* (in H1 haplotype) and *WAPO-A1b* (H2 haplotype) unique derived polymorphisms are indicated in red. “P−” indicates a 115-bp deletion in the promoter region and P+  = absence of this deletion. Negative coordinates are upstream of ATG and positive coordinates downstream (including intron). Amino acid changes are underlined and in bold and coordinates are from initial Met

In the first phase of the high-resolution mapping, we identified 74 plants showing recombination events among 617 F_2_ progeny screened with flanking markers IWB713 (670,767,495 bp) and IWB53096 (679,896,953 bp). Eleven additional KASP markers were developed across the target region (Supplementary File S2) based on SNPs identified in the exome-capture v2 data. We grouped the 74 recombinant HIF lines based on the location of the recombination events between the new markers and selected eleven recombinant lines for progeny tests. These lines were self-pollinated, and homozygous recombinant and non-recombinant sister lines were selected and evaluated for SNS in a greenhouse experiment. Using these phenotypic data and the KASP markers described above, the candidate region was reduced to 498 kb between markers Traes1400-I4V (673,779,017 bp, within *TraesCS7A02G481400*) and IWA5913 (674,276,906 bp) (Fig. 2a). We then screened 1208 additional segregating F_6:3_ plants with the new flanking markers but did not find additional recombination events in the reduced target region.

Homozygous recombinant and non-recombinant progeny of the four critical lines (F4, F7, F17 and F22) were reevaluated for SNS in a second greenhouse experiment, and the results of the two experiments were analyzed in a combined statistical analysis using experiments as blocks (Table 1). Significant differences in SNS were detected between recombinant and non-recombinant sister lines for families F4, F17 and F22 (classified as heterozygous) but not for family F7 (classified as homozygous, Table 1). The same results were obtained in an independent field experiment performed in 2018 using 10 replications per line (Fig. S2). Based on the replicated greenhouse and field experiments, the 7AL QTL was mapped as a single Mendelian locus within a 498-kb region delimited by markers Traes1400-I4V and IWA5913 (Table 1, Fig. 2a).

**Table 1:**
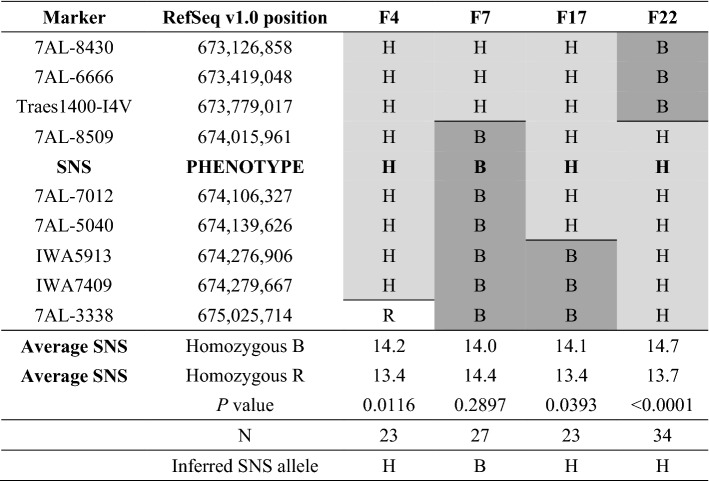
High-resolution map for the Berkut (B) × RAC875 (R) population based on progeny tests of lines showing the closest recombination events to the SNS locus

Physical positions on 7AL are based on RefSeq v1.0. Homozygous recombinant and non-recombinant sister lines were identified for each family using markers located within the segregating region (H). If significant differences were detected between sister lines in the combined ANOVA for the two greenhouse experiments, the SNS locus was mapped in the heterozygous region and if not in the homozygous region

Cells with an R and no shade indicate loci homozygous for the RAC875 allele, cells with an H and lighter gray shading indicate heterozygous loci and cells with a B and darker gray shade indicate loci homozygous for the Berkut allele

### High-resolution map of the SNS 7AL QTL in the Ning7840 × Clark population

The screening of 6496 progeny of HIFs segregating for 7AL QTL flanking markers IWB7435 and IWA5167 yielded 93 heterozygous recombination events. Based on the available markers in the region (Supplementary File S2), these recombinant lines were grouped into 31 recombinant classes. Their homozygous progenies were evaluated for SNS in a greenhouse experiment, and the 7AL QTL was mapped within a 423-kb region between markers AX-109397893 (673,854,124 bp) and IWA5913 (674,276,906 bp, Table 2). Additional markers were added (Supplementary File S2), and the ten lines with closest recombination events to the SNS locus were evaluated on a second greenhouse experiment. The combined analysis of the SNS data from the two greenhouse experiments for the five families with the closest recombination events is presented in Table 2. Families T13, T19 and T27 showed significant differences between homozygous recombinant and non-recombinant sister lines and were classified as heterozygous, whereas families T18 and T28 showed no significant differences and were classified as homozygous. Based on these statistical analyses and additional molecular markers described in Table 2, the 7AL QTL was mapped as a Mendelian locus within an 87-kb region between markers AX-111159341 (674,019,191 bp) and AX-109360122 (674,106,327 bp) (Table 5, Fig. 2a). An additional 3948 plants were screened with these new flanking markers, but no additional recombination events were detected in this small region. The candidate region identified in the N×C population was included within the 498-kb region identified in the B×R population (Fig. 2a).

**Table 2:**
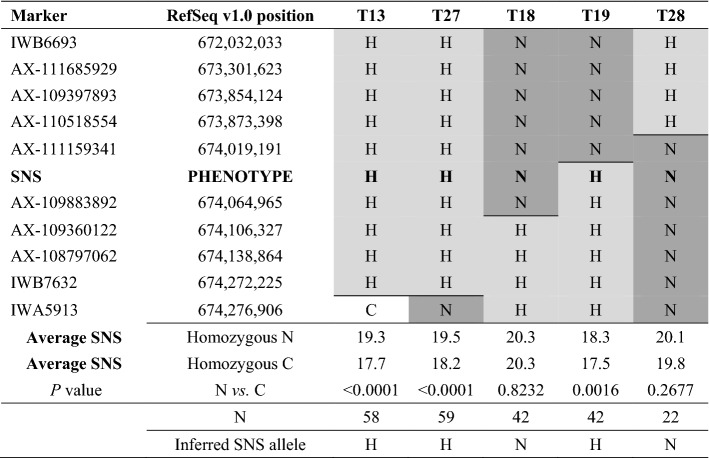
High-resolution map for the Ning7840 (N) × Clark (C) population based on progeny tests of lines showing the closest recombination events to the SNS locus

Physical positions on 7AL are based on RefSeq v1.0. Homozygous recombinant and non-recombinant sister lines were identified for each family using markers located within the segregating region (H). If significant differences were detected between sister lines in the combined ANOVA for the two greenhouse experiments, the SNS locus was mapped in the heterozygous region and if not in the homozygous region

Cells with a C and no shade indicate loci homozygous for the Clark allele, cells with an H and lighter gray shading indicate heterozygous loci and cells with an N and darker gray shade indicate loci homozygous for the Ning7840 allele

### Haplotype analysis

Analysis of the wheat exome-capture v2 data from 49 hexaploid common wheat cultivars and 4 tetraploid durum wheat cultivars (deposited in T3 https://triticeaetoolbox.org/wheat/) revealed three large haplotype blocks in the region (H1, H2 and H3, Fig. S3). The haplotype number does not reflect which haplotype is ancestral and which one is derived. These haplotype blocks span a 2.3-Mb region that extends approximately from 672.0 Mb to 674.3 Mb on chromosome arm 7AL. Haplotype H1 was identified in the parental lines associated with low SNS in the high-resolution maps described above (RAC875 and Clark) and included 13 hexaploid and 4 tetraploid lines (Fig. S3). Haplotype H2, which is present in the Chinese Spring reference genome, was detected in 33 hexaploid wheat lines including the two parental lines associated with high SNS in the high-resolution maps (Berkut and Ning7840, Fig. S3). Finally, the H3 haplotype was detected in three hexaploid lines from the South East of the USA (Fig. S3). We used SNP markers within this 2.3-Mb haplotype block to classify 238 additional lines from the photoperiod-insensitive spring wheat panel (Zhang et al. 2018) into haplotypes H1 (40 lines) and H2 (198 lines, Fig. S4). The H3 haplotype was not detected in this panel.

Using this information, we reevaluated the effect of SNPs located within and outside the 2.3-Mb haplotype block on SNS, kernel weight and total grain yield (Table 3). All markers included within the haplotype block showed highly significant differences in SNS, but *P* values dropped significantly for markers located 0.5 Mb proximal (IWA4911) or distal (IWA5961) to the haplotype block (Table 3). A combined analysis across the six environments where the GWAS panel was evaluated showed significant increases in SNS and decreases in kernel weight associated with the H2 haplotype, resulting in small (90.8 kg/ha) but significant increases in total grain yield (Table 3). This negative correlation between SNS and kernel weight was not observed in the B×R RILs. A combined ANOVA including the four environments where the B×R population was evaluated showed significant increase in SNS but no decrease in kernel weight, resulting in a larger (289.5 kg/ha) and more significant increase in total grain yield than in the GWAS (Table 3).

**Table 3:** Effect of SNPs located within and outside the 7AL haplotype block on spikelet number per spike (SNS), kernel weight (KW, in mg) and total grain yield (GY, in kg/ha)

SNP	RefSeq v1.0	*P* GWAS (6 env.)			*P* B×R RILs (4 env.)		
		SNS	KW	GY	SNS	KW	GY
IWA4911	671,417,177	3.56E−08	1.35E−11	6.01E−03	4.57E−12	0.2283	7.70E−03
IWB6693	672,032,033	2.08E−25	1.35E−05	0.1068	3.11E−11	0.3274	1.46E−02
IWB7632	674,272,225	6.20E−35	1.35E−04	1.74E−02	3.31E−10	0.3554	1.70E−02
IWA5912	674,276,849	4.61E−37	1.42E−06	1.34E−02	1.05E−11	0.3591	1.08E−02
IWA5913	674,276,906	4.61E−37	1.42E−06	1.34E−02	1.30E−11	0.3362	1.13E−02
IWA7409	674,279,667	1.20E−32	2.18E−04	2.76E−02	2.23E−10	0.2735	4.50E−03
IWB5961	674,801,909	3.79E−01	2.13E−01	6.49E−01	1.30E−11	0.3362	1.13E−02
Berkut (H2 haplotype)^a^		20.7 ± 0.03	33.6 ± 0.07	4441 ± 16	19.8 ± 0.2	55.2 ± 1.6	4431 ± 77
RAC875 (H1 haplotype) ^a^		19.7 ± 0.06	34.3 ± 0.15	4350 ± 33	17.7 ± 0.2	52.9 ± 1.7	4141 ± 83
H2 − H1		0.9	− 0.8	90.8	2.10	2.2	289.5
(H2 − H1)/H1		4.7%	− 2.3%	2.1%	11.8%	4.2%	7.0%

The GWAS analysis included 262 photoperiod-insensitive spring wheats evaluated in six environments in CA and the B×R population 75 segregating RILs in four environments (see "Materials and Methods"). *P* values in the body of the table are from ANOVAs for each marker using environments as blocks

^a^ Averages (least-square means) and standard errors of the means were calculated from the ANOVA using environments as blocks and marker IWA5913 as classification variable

The wheat accessions genotyped with the wheat exome-capture v2 assay included parental lines of 15 mapping populations used in the USDA/IWYP-WheatCAP project to characterize QTLs affecting grain yield (https://www.triticeaecap.org/qtl-cloning-projects/). Using this resource, we identified additional populations segregating for different haplotypes (Table 4). Three of these populations (one tetraploid and two hexaploid) segregated for the H1 and H2 haplotypes, and in all three populations the H2 haplotype was associated with a highly significant increase in SNS relative to the H1 haplotype (*P* < 0.0001, Table 4). Interestingly, the LA95135 × MPV57 population segregated for a different haplotype combination (H2 × H3). In this population, the H2 haplotype was associated with significantly higher SNS than the H3 haplotype. This result was important because it showed that the H2 haplotype conferred a higher SNS than both H1 and H3, which allowed us to focus on SNPs that were polymorphic between H2 and both H1 and H3.

**Table 4:** Additional four recombinant inbred line (RIL) populations validated for the 7AL SNS QTL using marker IWA5913 within the haplotype block

Population	Market class ^a^	Haplotype	*N*	Evaluation	*P* value SNS ^b^
Ben × PI 41025	Tetraploid	H1x H2^c^	197 F_7:8_	Prosper, ND	*P* < 0.0001
MN98550-5 × MN99394-1	HRS (6x)	H2^c^ × H1	138 F_6:9_	Crookston, MN	*P* < 0.0001
CO-940610 × Platte	HWW (6x)	H1 × H2^c^	223 F_5:6_	Fort Collins, CO	*P* < 0.0001
LA95135 × MPV57	SRW (6x)	H3 × H2^c^	324 F_6:7_	Raleigh/Kinston, NC	*P* < 0.0001

^a^Tetraploid: Ben is a durum wheat and PI 41025 a cultivated emmer. *HRS* hard red spring, *HWW* hard white winter, *SRW* soft red winter

^b^ For populations evaluated in more than one location or more than one replication (ND, CO, NC), environments were used as blocks in a combined ANOVA to calculate the reported *P* values

^c^ In all experiments, the H2 haplotype was associated with a significant increase in SNS

### Candidate genes

The 87-kb candidate gene region determined by the high-resolution map includes two complete genes, *TraesCS7A02G481600* and *TraesCS7A02G481700*, and the 3′ region of *TraesCS7A02G481500* and *TraesCS7A02G481800* (Fig. 2a, Table 5). The promoter and 5′ coding regions of the last two genes were excluded from the candidate region by recombination (Fig. 2a). Below we discuss the four candidate genes following their chromosome order.

**Table 5:** Predicted changes in proteins encoded by polymorphic high-confidence genes annotated in the candidate gene regions defined by the B×R and N×C flanking markers (underlined)

Gene name	RefSeq v1.0	Berkut (H2) vs RAC875 (H1)	MPV57 (H2) vs LA95135 (H3)	Protein annotation	
*TraesCS7A02G481400*	673,779,017	**I4V**	**I4V**	CYTOCHROME C OXIDASE SUBUNIT 6A	
B×R Proximal marker	673,779,017				
*TraesCS7A02G481500*	674,018,426	Not polymorphic	**R207G**	AMIDOHYDROLASE	
N×C Proximal marker	674,019,191				
*TraesCS7A02G481500*	674,019,603	Synonymous	Not polymorphic		
*TraesCS7A02G481500*	674,019,951	Synonymous	Synonymous		
*TraesCS7A02G481500*	674,020,022	**V456A**	Not polymorphic		
*TraesCS7A02G481600*	674,080,862^a^	**P+/P− (115 bp ** **del)**	Not polymorphic	ABERRANT PANICLE ORGANIZATION 1	
*TraesCS7A02G481600*	674,081,601	**C47F**	**C47F**		
*TraesCS7A02G481600*	674,082,745	**D384N**	Not polymorphic		
*TraesCS7A02G481700*	674,091,310	Synonymous	Not polymorphic	MAJOR POLLEN ALLERGEN OLE E 10-LIKE	
*TraesCS7A02G481700*	674,091,312	Not polymorphic	**L177H**		
N×C Distal marker	674,106,327				
*TraesCS7A02G481800*	674,106,471	Synonymous	Not polymorphic	SYNTAXIN	
*TraesCS7A02G481900*	674,115,185	Not polymorphic	**E46A**	CASP-LIKE PROTEIN 5B3	
*TraesCS7A02G482000*	674,272,225	E252*	Not polymorphic	HYDROLASE	
*TraesCS7A02G482100*	674,274,282	Synonymous	Not polymorphic	DETOXIFICATION 34-LIKE	
*TraesCS7A02G482100*	674,274,822	Not polymorphic	Synonymous		
B×R Distal Marker	674,276,883				

^a^ Start of the 115-bp deletion in the promoter

*TraesCS7A02G481500*, a gene annotated to encode an AMIDOHYDROLASE, showed a non-synonymous substitution (V456A, BLOSUM 62 score = 0) in the exons located within the candidate region. This polymorphism was present in the B × R (H2 × H1) population but not in the LA95135 × MPV57 population (H3 × H2) (Table 5) and therefore cannot explain the differences in SNS detected in both populations.

*TraesCS7A02G481600* is an ortholog of the rice gene *APO1*, which is known to affect the number of spikelets in the panicle (Ikeda et al. 2007). The proteins encoded by the rice and wheat genes are 82.5% identical (87% similar) over 93% of their length suggesting a conserved function. *TraesCS7A02G481600* is designated here as *WHEAT ORTHOLOG OF APO1* (*WAPO1*), and the three homoeologs as *WAPO-A1* (*TraesCS7A02G481600*, Fig. 2a, b), *WAPO-B1* (*TraesCS7B02G384000*) and *WAPO-D1* (*TraesCS7D02G468700*). *WAPO-A1* is the only gene within the 7AL QTL candidate region with an amino acid change (C47F) that differentiates H2 from both H1 and H3 haplotypes (Table 5). The C47F polymorphism is located within the conserved F-box (Fig. 2b, amino acids 36–74) and has a negative BLOSUM 62 score -2, indicative of a disruptive amino acid change (Table 5). The C47 allele was detected in *WAPO-B1* and *WAPO-D1* and in the other 8 grass genera analyzed in this study. By contrast, the F47 allele was detected in the 46 non-grass genera included in the analysis (Fig. S5).

The only other complete gene within the candidate gene region is *TraesCS7A02G481700*, which encodes a protein containing an X8 domain (pfam07983, BLASTP E-value = 1.46 e^−24^). This domain is found at the C-terminus of several families of glycosyl hydrolases and in an Olive pollen allergen (MAJOR POLLEN ALLERGEN OLE E 10-LIKE). The encoded protein is thought to be involved in carbohydrate binding. This gene showed an L177H amino acid difference between LA95135 and MPV57, but not between Berkut and RAC875, suggesting that it cannot explain the differences in SNS detected in both populations (Table 5). In the first 1,500 bp upstream of the start codon of gene *TraesCS7A02G481700*, we detected four SNPs that differentiated H2 from both the H3 and H1 haplotypes. However, the alignment of wheat, barley, *Brachypodium* and rice *APO1* promoters showed that none of these four SNPs were within conserved regions. A search in a database of plant transcription factor binding site (Solovyev et al. 2010) also showed that these four SNPs were not within conserved binding sites. Although these results reduce the likelihood that these four SNPs play a critical role in the differences in SNS, we cannot completely rule out this gene as a candidate underlying the SNS QTL.

The last gene in the candidate region, *TraesCS7A02G481800*, encodes a SYNTAXIN, a member of the SNARE family of proteins involved in ER-Golgi transport and Golgi-endosome transport. The promoter of this gene is excluded from the candidate region, and the ten exons within the candidate regions show no polymorphisms between the parental lines of the different mapping populations. In summary, among the four genes located within the candidate gene region, the genetic evidence and the annotated gene functions point to *TraesCS7A02G481600* as the most likely candidate gene for the SNS QTL.

### Natural variation in *WAPO-A1*

#### Sequenced accessions

Table 6 provides a summary of the SNPs detected in the *WAPO-A1* genomic sequence obtained from published wheat genomes and from several accessions sequenced in this study (see Materials and Methods). Henceforth, we will use the term ‘allele’ when referring to the sequence variants of *WAPO-A1* and the term ‘haplotype’ when referring to the linked SNPs in the 87-kb candidate region. Following the wheat gene nomenclature, alleles will be designated by a lowercase letter after the gene name.

**Table 6:** Natural variation in genomic sequences of *WAPO-A1*

Haplotype in the candidate region			Haplotype H1	Haplotype H2	Haplotype H3		
RefSeq v1.0	DNA change^a^	Effect	*WAPO-A1a*	*WAPO-A1b*	*WAPO-A1c*	*WAPO-A1d*	Consensus
*DNA change*							
674,080,862	− 599/− 484	Del. Pro	**Yes**	No	No	No	No
674,081,002	G-460A	Promoter	G	**A**	G	G	G
674,081,218	A-244G	Promoter	A	**G**	A	A	A
674,081,328	G-134T	Promoter	G	**T**	G	G	G
674,081,396	C-66T	Promoter	C	C	C	**T**	C
674,081,858	C397T	Syn	C	C	**T**	C	C
674,082,225	G764A	Intron	G	G	G	**A**	G
674,082,248	C787T	Intron	C	C	**T**	C	C
674,082,303	T842C	Intron	T	**C**	T	T	T
674,082,435	C974G	Syn	C	C	**G**	C	C
*Amino acid change* ^b^							
674,081,601	G140T	C47F	C	**F**	C	C	C
674,082,745	G1284A	D384N	**N**	D	D	D	D

The accessions sequenced for each allele are listed in Table S2

^a^First letter indicates ancestral nucleotide and numbers distances from start codon in genomic sequence including the intron. Derived unique alleles are in bold

^b^Numbers in the amino acid changes correspond to the position in the predicted protein from the starting Met. C47F is the only amino acid change within the conserved F-box domain (amino acids 36–74)

The *WAPO-A1a* allele detected in haplotype H1 differed from all other alleles by a 115-bp deletion located − 599 to − 485 upstream of the start codon (henceforth referred to as P−) and a D384N amino acid change (P−/C47/N384) (Fig. 2b, c, Table 6). We sequenced the complete gene in the parental lines RAC875, Clark, Ben, CO-940610 and MN99394-1 and confirmed the presence of identical *WAPO-A1a* alleles (Tables 4 and S2). The *WAPO-A1b* allele detected in haplotype H2 differed from all other alleles by the C47F amino acid change (P+/F47/D384) and by a C842 SNP in the intron and three SNPs in the promoter region (G-460A, A-244G, and G-134T, Table 6). By Sanger sequencing, we confirmed the presence of *WAPO-A1b* identical alleles in the parental lines Berkut, Ning7840, PI 41025, MPV57, Platte and MN98550-5 (Tables 4 and S2).

Haplotype H3 has the *WAPO-A1* combination P+/C47/D384, which was found in the diploid donor of the A genome (*T. urartu*), the *WAPO-B1* and *WAPO-D1* homoeologs, and other grass species and therefore was considered the ancestral combination. Among the sequenced polyploid wheat alleles with the P+/C47/D384 combination, *WAPO-A1c* differed from all other alleles by linked SNPs C397T (synonymous), C787T (intron) and C974G (synonymous, Table 6). The *WAPO-A1c* allele was detected in wild and cultivated emmer wheats, in cultivated tetraploid and hexaploid wheats and in spelt wheats (*T. aestivum* subsp. *spelta*, Supplementary Table S3). *WAPO-A1d* differed from other alleles by a G764A (synonymous) and C-66T (upstream of the start codon, Table 6) polymorphisms. The *WAPO-A1d* allele was found in two durum wheats (Rusty and Langdon) and nine cultivated emmer wheats (Table S2). Table 6 also presents the consensus among these four alleles, which can be defined by the combination P+/C47/D384 and likely represents the ancestral state.

#### Markers for C47F and promoter deletion

We designed PCR markers for the 115-bp promoter deletion and the C47F polymorphisms that were sufficient to differentiate the *WAPO-A1a* and *WAPO-A1b* from the other alleles (P+/C47/D384). The PCR primers for the promoter deletion (Supplementary File S2) amplified a fragment of 210 bp from the accessions carrying the *WAPO-A1a* allele (P−) and a fragment of 325 bp in those carrying other alleles (P+). For the C47F polymorphism, digestion of the amplification products obtained with dCAPS primers (Supplementary File S2) with restriction enzyme *Hpy*CH4V yielded a fragment of 200 bp for the F47 allele and a fragment of 180 bp for the C47 allele.

**Fig. 3:**
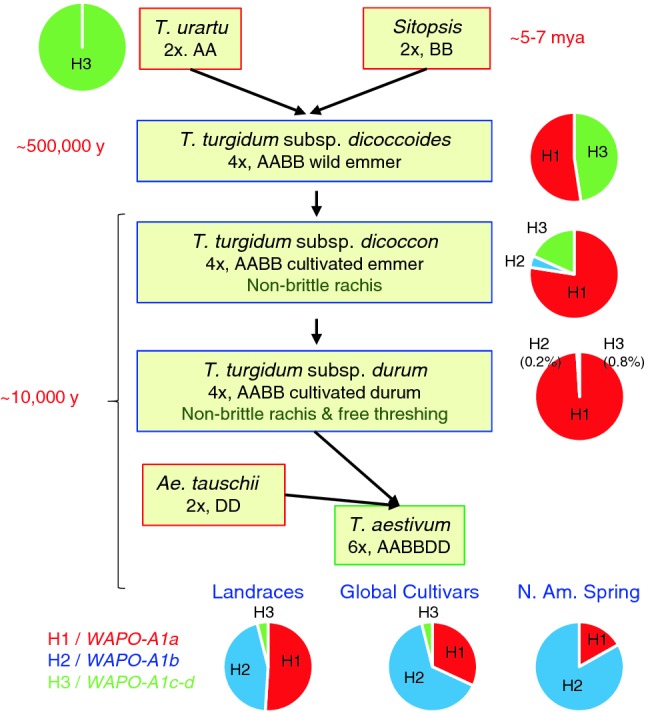
Frequency of alleles *WAPO-A1a* (P-/C47/N384, haplotype H1), *WAPO-A1b* (P+/F47/D384, haplotype H2) and *WAPO-A1c-d* (P+/C47/D384, haplotype H3) in different wild and cultivated species and subspecies of wheat. Accessions used to estimate allele frequencies are summarized in Supplementary File S3. The hexaploid wheats were separated in three groups: old landraces, worldwide diverse collection of old and new improved cultivars (He et al. 2019), and a spring wheat panel including mainly modern cultivars from North America and CIMMYT (Zhang et al. 2018)

Using these primers, we characterized a collection of wild and cultivated wheat accessions described in Supplementary File S3. The P−/C47 marker combination (*WAPO-A1a*) was not detected in any of the 74 T*.urartu* accessions tested, but was frequent in wild emmer (52.4%). The frequency of this allele increased to 77.5% in cultivated emmer and was almost fixed in cultivated durum (98.9%, Fig. 3). By contrast, in a subset of 238 modern spring common wheat cultivars (Supplementary File S3), the *WAPO-A1a* allele was found in only 16.8% of the accessions (Fig. 3). The P+/F47 marker combination (*WAPO-A1b*) was not detected in *T. urartu* or wild emmer and was found only in three accessions of cultivated emmer from Ethiopia and Russia and one durum accession from Syria (Supplementary File S3). By contrast, this allele was found in 83.2% of the common wheats included in the panel of modern spring wheat cultivars (Fig. 3, Supplementary File S3).

The ancestral P+/C47 marker combination was detected in all tested accessions of *T. urartu,* in 47.6% of the analyzed wild emmer wheats and in 18.6% of the cultivated emmer accessions (Fig. 3, Supplementary File S3). However, the frequency of this marker combination decreased sharply in modern cultivated wheat and was found in 4% of the hexaploid wheat worldwide collection of landraces and cultivars, 0.8% of the cultivated durum, and 0% in the panel of modern spring common wheat cultivars (Fig. 3, Supplementary File S3).

#### Exome capture

We also explored the *WAPO-A1* region in a diverse worldwide collection including 813 hexaploid wheat accessions (He et al. 2019) genotyped with the wheat exome-capture assay v1 (Jordan et al. 2015). Supplementary File 3 presents six polymorphisms within *WAPO-A1* and the 13 closest SNPs covering a region of 63 kb. (Accessions with > 10 missing genotype calls within this region were excluded.) This analysis included only accessions that were classified as landraces (257 accessions) or improved cultivars (387 accessions), to compare the frequencies of the *WAPO-A1* alleles between both groups. Landraces and improved cultivars showed similar frequencies of the *WAPO-A1c* allele (4%) but differed in the *WAPO-A1a* and *WAPO-A1b* frequencies. In the global wheat collection, the frequency of the *WAPO-A1a* allele was significantly higher in common wheat landraces (51.0%) than in the improved cultivars (34.4%, homogeneity χ^2^ test *P* = 0.0034), and the opposite trend was observed for the *WAPO-A1b* allele (45.1% in landraces and 62.5% in improved cultivars, Fig. 3). The difference between the *WAPO-A1b* (83.2%) and *WAPO-A1a* (16.8%) allele frequencies was even higher in the spring wheat panel than in the global cultivar and landrace collection (homogeneity χ^2^ test *P* < 0.0001, Fig. 3, Supplementary File 3).

The exome-capture study included different *T. aestivum* subspecies¸ which are summarized in Supplementary File 3 together with the exome-capture data. The three *T. aestivum* subsp. *macha* and the five *T. aestivum* subsp. *sphaerococcum* accessions (including two analyzed with the promoter and C47F markers) all carried the *WAPO-A1a* allele, whereas the three *T. aestivum* subsp. *compactum* accessions carried the *WAPO-A1b* allele. Among the 15 accessions of *T. aestivum* subsp. *spelta* analyzed here, eight carried *WAPO-A1c*, six *WAPO-A1b* and one *WAPO-A1a* (Supplementary File S3).

In summary, the analysis of *WAPO-A1* natural variation revealed rapid increases in the frequency of *WAPO-A1a* in durum and of *WAPO-A1b* in common wheat varieties.

### Expression of candidate genes

**Fig. 4:**
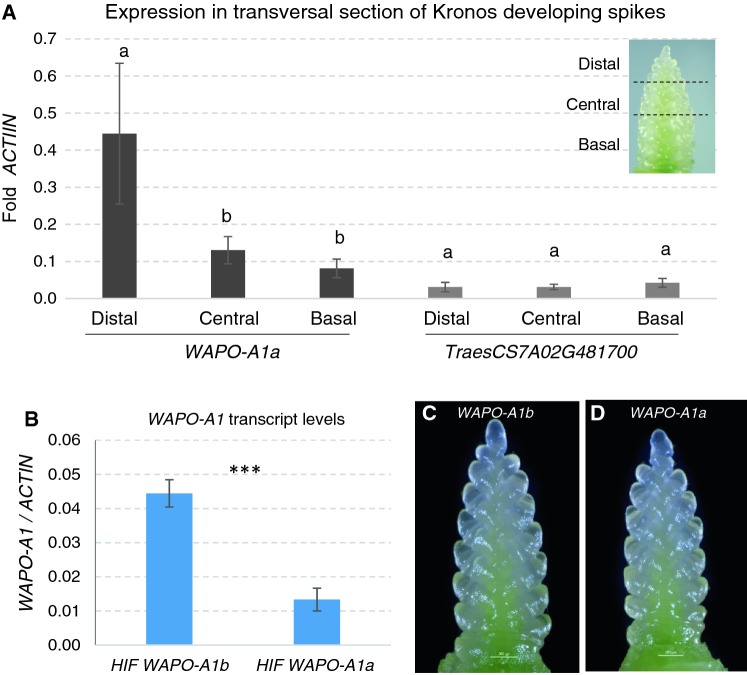
Transcript levels in developing spikes at the floret primordia stage relative to *ACTIN* as endogenous control. **a***WAPO-A1* and *TraesCS7A02G481700* in basal, central and distal sections of Kronos developing spikes. The experiment was repeated twice, and data were analyzed together using experiment as block. Means within each gene were compared using Tukey tests ( = 0.05). Bars are SE of the means. **b** Transcript levels of *WAPO-A1* in hexaploid near isogenic HIF lines homozygous for the *WAPO-A1b* and *WAPO-A1a* alleles. **c–d** Representative spikelet of the HIFs carrying the **c***WAPO-A1b* (Berkut) allele or **d** the *WAPO-A1a* (RAC875) allele. Four replications were analyzed per genotype, each consisting on pooled developing spikes at the same developmental stage from 9 to 12 plants

For the two genes that have their promoters within the candidate gene region, *WAPO-A1* and *TraesCS7A02G481700* (Fig. 2), we compared transcript levels by qRT-PCR in developing spikes at the floret primordia stage from Kronos (*WAPO-A1a* allele, promoter deletion/C47). Since SNS is determined by the timing of the transition of the distal inflorescence meristem into a terminal spikelet, we also compared *WAPO-A1* expression in basal, central and distal sections of spikes at the spikelet development stage (Fig. 4a). *WAPO-A1a* showed significantly higher transcript levels in the distal region than in the central or basal region (Tukey test *P* < 0.05, Fig. 4a), whereas *TraesCS7A02G481700* showed lower transcript levels than *WAPO-A1* and no significant differences among the three regions (Fig. 4a). More precise in situ hybridization experiments will be necessary to determine whether the higher expression of *WAPO-A1* in the distal region is due to increased expression in the inflorescence meristem and/or in the younger lateral meristems included in the distal section.

We then compared *WAPO-A1* transcript levels in developing spikes at the floret primordia stage between HIF lines homozygous for the *WAPO-A1b* (Berkut) and the *WAPO-A1a* allele (RAC875). Transcript levels of *WAPO-A1* in plants homozygous for the Berkut allele (*WAPO-A1b*) were more than threefold higher (*P* < 0.0006) than in plants homozygous for the RAC875 allele (*WAPO-A1a*, Fig. 4b). This result suggests that either the 115-bp deletion in the *WAPO-A1a* promoter reduces its transcript levels, or the three unique SNPs in the *WAPO-A1b* promoter (Table 6) increase its expression. Although the reduced expression of *WAPO-A1a* may contribute to the reduced SNS, this experiment cannot separate the effects of the differences in transcript levels from the effect of the amino acid differences between the encoded proteins (C47F and D384N).

## Discussion

### Identification of a candidate gene for the 7AL QTL for SNS

The haplotype analysis of the SNS QTL region on chromosome arm 7AL (Fig. S3) revealed the existence of a large haplotype block of approximately 2.3 Mb. This haplotype block was also detected in a panel of soft red winter wheats (Ward et al. 2019), several panels of European winter wheats (Boeven et al. 2016; Voss-Fels et al. 2019; Wurschum et al. 2018), and the panel of USA and CIMMYT photoperiod-insensitive spring wheats included in this study (Fig. S4). In the absence of historical recombination events, the GWAS for SNS showed similar levels of significance for SNPs located within the 2.3-Mb haplotype block (Table 3). This result highlighted the limitations of GWAS panels including only modern wheat cultivars for the identification of a specific candidate gene within this region. The use of the more diverse panel of accessions included in the large exome-capture study (He et al. 2019; Jordan et al. 2015) revealed several historical recombination events within this 2.3-Mb haplotype block, which were also detected in the companion paper (Voss-Fels et al. 2019).

The two large biparental populations used in this study partitioned the 2.3-Mb block and reduced the candidate gene region to 87 kb (Fig. 2). Among the four genes annotated in this region (Table 5), only *WAPO-A1* (*TraesCS7A02G481600*) showed a non-synonymous polymorphism consistent with the significant differences in SNS detected between H2 (F47) and both H1 and H3 haplotypes (C47, Table 4). This result, however, is not sufficient to rule out the other three genes (*TraesCS7A02G481500*, *TraesCS7A02G481700* and *TraesCS7A02G481800,* Table 5) as candidates for the SNS QTL, because polymorphisms in regulatory regions not included in this study may explain the differences in SNS observed between H2 and both H1 and H3 haplotypes. This possibility is less likely for *TraesCS7A02G481500* and *TraesCS7A02G481800* because their promoter regions are outside the candidate gene region (Fig. 2). The *TraesCS7A02G481700* promoter is within the candidate gene region and includes four SNPs that differentiate H2 from both H1 and H3. However, none of these SNPs were within conserved regions of the promoter nor within known transcription factor binding sites, reducing their chances of playing critical roles in the transcriptional regulation of these genes.

The annotated functions of the proteins encoded by *TraesCS7A02G481500*, *TraesCS7A02G481700* and *TraesCS7A02G481800* seem unrelated to known genes involved in the regulation of inflorescence development. *TraesCS7A02G481500* encodes an AMIDOHYDROLASE enzyme, *TraesCS7A02G481700* encodes an X8-domain protein, with similarity to the C-terminus of several families of GLYCOSYL HYDROLASES, and *TraesCS7A02G481800* encodes a SYNTAXIN. These annotated gene functions have not been associated previously with inflorescence morphology or regulation of changes in meristem identity. By contrast, *WAPO1* is orthologous to *APO1*, a rice gene known to affect the number of spikelets per panicle (Ikeda-Kawakatsu et al. 2009), a phenotype that is consistent with the effect of the 7AL QTL in wheat.

Expression profiles of *WAPO-A1* are also consistent with the postulated role of this gene in the regulation of SNS. *WAPO-A1* transcript levels were higher in the distal third of the developing wheat spike, a region that is critical for the determination of SNS. This observation is consistent with in situ hybridization results in rice, which showed expression of *APO1* in the inflorescence meristem and lateral primordia at early stages of panicle development (Ikeda-Kawakatsu et al. 2009).

Loss-of-function mutations in the *apo1* rice mutants have a precocious transition of the inflorescence meristem into a terminal spikelet, resulting in a reduced number of branches and spikelets than the wild type (Ikeda et al. 2007), whereas rice lines carrying alleles with increased *APO1* expression showed more branches and spikelets per panicle than the wild type (Ikeda et al. 2007). This result is similar to what we observed in wheat, where lines carrying the *WAPO-A1b* allele showed higher transcript levels and higher SNS than isogenic lines carrying the *WAPO-A1a* allele (Fig. 4b). Although this result suggests that differences in *WAPO-A1* expression can contribute to the observed differences in SNS, we cannot rule out the possibility that these differences were caused by the different amino acids present in the proteins encoded by these two alleles. We are currently developing isogenic lines to compare *WAPO-A1* expression levels in different allele combinations.

In summary, the strong genetic evidence, the known function of the rice *APO1* ortholog on the regulation of spikelet number, and the preliminary expression results, all point out to *WAPO-A1* as the best candidate gene for the 7AL SNS QTL among the four genes detected in the 87-kb candidate region. Transgenic complementation and mutant analysis are still pending for a formal demonstration of causality.

### Natural variation in *WAPO1 *in wild and cultivated wheat

The characterization of *WAPO-A1* allelic variation provided interesting insights, but also opened several questions. One of these questions is the effect of the C47F amino acid change in the conserved F-box, which differentiated the high-SNS allele *WAPO-A1b* from the low-SNS alleles *WAPO-A1a* and *WAPO-A1c*. The C47 allele is most likely the ancestral allele in wheat because it is fixed in the diploid donor of the A genome (*T. urartu*), WAPO-B1 and WAPO-D1, and in the predicted WAPO1 proteins from the eight additional grass genera investigated (Fig. S5). By contrast, the F47 variant was fixed in all 46 non-grass genera investigated here (Fig. S5). No amino acids other than C or F were found at this position in any of the plant species investigated so far. Based on its negative BLOSUM 62 score (-2) and its location within the conserved F-box, this amino acid change is likely to have an effect on protein structure and/or function. We hypothesize that the C47 and F47 alleles are both functional and have different effects on SNS. Knockout mutations of this gene in both rice (C47) (Ikeda et al. 2007) and Arabidopsis (F47) (Wilkinson and Haughn 1995) have strong phenotypic effects, indicating that they are not null alleles. However, we cannot rule out the possibility that the F47 change in *WAPO-A1b* has no effect on SNS in wheat because this allele also differs from *WAPO-A1a* and *WAPO-A1c* by three SNPs in the promoter region. We have initiated transgenic experiments including the C and F variants to test these alternative hypotheses.

We did not find the F47 amino acid (*WAPO-A1b*) in the 21 T*. turgidum* subsp. *dicoccoides* analyzed in this study (Supplementary File S3), but the H2 haplotype was detected in 1% of the wild emmer wheat accessions in the companion paper (Voss-Fels et al. 2019). The frequency of the *WAPO-A1b* allele was low in cultivated emmer and cultivated durum, but was substantially higher in hexaploid wheat where we observed a rapid increase from 45.1% in the landraces to 83.2% in the modern cultivars (Fig. 3). This rapid change suggests that *WAPO-A1b* might have been favored by positive selection, likely due to its positive effect on SNS and grain yield (Table 3). However, we cannot rule out the possibility that selection for other genes within the H2 haplotype contributed to its increased frequency in hexaploid wheat.

The decrease in *WAPO-A1a* allele frequency in hexaploid wheat (Fig. 3) contrasts with its rapid increase in tetraploid wheat, where it increased from 52.4% in wild emmer to 98.9% in durum cultivars (Fig. 3). Similar trends were reported in the companion paper (Voss-Fels et al. 2019). We currently do not know whether the high frequency of the *WAPO-A1a* in tetraploid wheat is the result of a random fixation due to the low frequency of the *WAPO-A1b* allele in ancestral tetraploid wheats, or the result of indirect selection for larger grains in tetraploid wheat. Under the latter scenario, a strong selection for large grains in durum wheat could have driven an indirect selection for reduced SNS, and favored the *WAPO-A1a* over the *WAPO-A1b* allele in tetraploid wheat.

Finally, the rapid increases in the frequency of the *WAPO-A1b* allele in common wheat and the *WAPO-A1a* allele in durum wheat likely explain the displacement of the ancestral *WAPO-A1* allele (P+/C47). The frequency of the ancestral allele was relatively high in wild emmer (47.6%), decreased in cultivated emmer (18.8%) and is currently very low in modern cultivated durum (< 1%) and common wheat (< 5%).

### Effect of the different haplotypes on SNS, kernel weight and total grain yield

The significant increases in SNS associated with the H2 haplotype in both tetraploid and hexaploid wheats, in different common wheat market classes and in contrasting environments (Table 4) suggest that this haplotype may have a broad beneficial effect on grain yield. However, the beneficial H2 haplotype is already present at a high frequency in most of the spring wheat breeding programs in the USA and CIMMYT (77–93%) (Zhang et al. 2018), so only a small proportion of these programs can benefit from a more systematic selection for H2. By contrast, H2 is almost absent in durum wheat so it can have a beneficial effect in a broader germplasm base if it can be confirmed to improve grain yield when introgressed in a modern durum background. We are currently introgressing H2 into commercial durum cultivars to test its effect on total grain yield.

The results presented in Table 3 are informative of what can be expected from the replacement of the H1 by H2 in common wheat in different environments. In the GWAS, the potential gains obtained by the increase in SNS (4.7%) were partially offset by a significant decrease in average grain weight (-2.3%), resulting in a grain yield increase of only 2.1% (90.8 kg/ha, Table 3). By contrast, the significant increase in SNS (11.8%) in the B×R RILs carrying the H2 haplotype was not associated with a negative effect on kernel weight. As a result, we observed a much larger increase in total grain yield (289.5 kg/ha = 7.0%, Table 3) in the B×R RILs than in the GWAS. The increases in grain yield in the 7AL QTL region in both populations were not associated with significant differences in the normalized difference vegetation index, suggesting a limited effect of these alleles on vegetative growth or biomass.

Taken together, these results suggest that the better adapted B×R RILs had sufficient resources (‘source’) to fill the extra grains associated with the H2 haplotype, whereas the less adapted lines from the GWAS were not able to do it, resulting in a negative correlation between grain number and grain size. This is not surprising, considering that 38% of the lines in the spring panel were developed in spring planting regions and were not well adapted to the typical fall planting conditions used in the Mediterranean climate of California (Zhang et al. 2018).

Environmental conditions can also affect the proportion of the increases in SNS that are translated into increases in total grain yield. In each of the two locations where the B×R population was analyzed (Davis and Imperial Valley), the RILs were grown either under full irrigation or under terminal drought (irrigation interrupted after booting). The increase in total grain yield in H2 relative to H1 RILs was higher in the irrigated treatments (9.7%) than in the terminal drought treatments (3.6%). A similar trend was observed between the two terminal drought treatments, with a larger increase in total grain yield in the milder climate of Davis (7.3%) than in Imperial Valley ( = 0.5%), where higher temperatures amplified the effect of the reduced irrigation. We suggest that the higher temperatures and more severe terminal drought in the Imperial Valley relative to Davis reduced the plant growth (source) and limited the ability of the plants to translate the increases in SNS into increases in total grain yield. A large impact of the environment on the effect of the H2 and H1 haplotypes on grain yield was also observed in the companion paper (Voss-Fels et al. 2019).

In summary, our results suggest that the proportion of the beneficial increases in SNS (sink) associated with the H2 haplotype that are translated into increases in total grain yield depends on the genotypes and the environments where these genotypes are grown. Genotypes with no or low limitation in the source (e.g., higher biomass and good harvest indexes) grown in environments with abundant water and fertilizer will likely translate a higher proportion of the increases in SNS into increases in total grain yield. As our understanding of the wheat genes controlling grain number (Alvarez et al. 2016; Dixon et al. 2018; Li et al. 2019; Poursarebani et al. 2015; Sakuma et al. 2019; Shaw et al. 2019; Wolde et al. 2019) and grain weight (Simmonds et al. 2016) improves, additional research will be required to understand the genes controlling the ‘source’ (e.g., growth and biomass) to generate a balanced increase in wheat grain yield potential.

#### Author contribution statement

SK, YX and JZ conducted most of the experimental work. ZS contributed to the high-resolution maps, and AK, SP, MF, JA, GBG, ND, APH and JDF contributed additional mapping populations. EA and JZ performed exome capture and did haplotype and selection studies; GB and JD initiated and coordinated the project, contributed to data analyses and wrote the final manuscript. All authors reviewed the manuscript and provided suggestions.

## Electronic supplementary material

Below is the link to the electronic supplementary material.

Supplementary file1 (XLSX 240 kb)

Supplementary file2 (XLSX 477 kb)

Supplementary file3 (XLSX 22 kb)

Supplementary file4 (PDF 171 kb)
