# Supplementary material for: Identification of a candidate gene for a QTL for spikelet number per spike on wheat chromosome arm 7AL by high-resolution genetic mapping
Source: Theor Appl Genet. 2019 Jun 28;132(9):2689–705. doi: 10.1007/s00122-019-03382-5 (PMC6708044; doi:10.1007/s00122-019-03382-5)
Supplement: Supplementary file 4 — Supplementary file4 (PDF 171 kb) [file 122_2019_3382_MOESM4_ESM.pdf]

Supplementary Figures

Figure S1. Evaluation and characterization of recombinant inbred lines (RILs) and heterogeneous inbred families (HIFs) developed from heterozygous Berkut x RAC875 RILs 23 and 42. **A**) Differences in spikelet number per spike (SNS) in HIFs carrying the Berkut or the RAC875 alleles for the 7AL QTL. **B**) Number of replications required to detect significant differences in SNS between the Berkut and RAC875 alleles in RIL and HIF populations evaluated in field experiments at Davis and Imperial Valley (California, USA). The number of replications are calculated for a statistical power of 0.9 and two levels of alpha (0.05 and 0.01). Note the lower pooled standard deviation and replications required in the HIFs relative to the RILs.

A

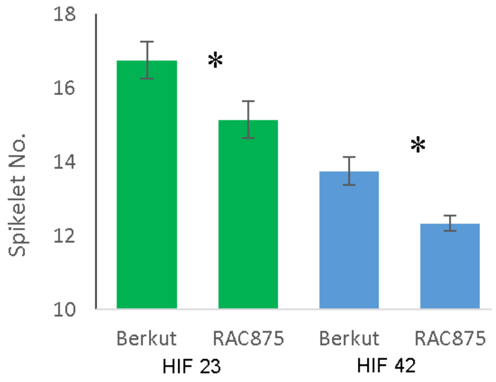

B

7AL marker IWA5913	Field Exp.	Mean	Mean Dif.	Pooled std. dev.	Power 0.90	
					α= 0.05	α= 0.01
Berkut allele<br>RAC875 allele	RILs Davis	19.91<br>17.98	1.93	2.03	25	35
Berkut allele<br>RAC875 allele	RILs Imp.	19.51<br>17.63	1.88	2.08	27	39
Berkut allele<br>RAC875 allele	HIFs Davis	20.57<br>19.35	1.22	**0.56**	**6**	**9**

Figure S2. Inferred genotypes of the SNS gene in critical recombinant lines from Berkut x RAC875 HIF identified in the high-resolution map. Homozygous recombinant lines from families F4, F7, F17 and F22 and parental HIF 42 lines carrying the Berkut (B) and RAC875 (R) alleles were planted as one meter rows in a randomized complete block design with 10 blocks. Three spikes were measured and averaged per row. SNS in each recombinant line was statistically compared to the average of all other lines carrying the RAC875 and Berkut alleles.

Marker	RefSeq v1.0	F4	F7	F17	F22
7AL-8430	673,126,858	B	R	R	B
7AL-6666	673,419,048	B	R	R	B
Traes1400-I4V	673,779,017	B	R	R	B
7AL-8509	674,015,961	B	B	R	R
SNS	**PHENOTYPE**	**B**	**B**	**R**	**R**
7AL-7012	674,106,327	B	B	R	R
7AL-5040	674,139,626	B	B	R	R
IWA5913	674,276,906	B	B	B	R
IWA7409	674,279,667	B	B	B	R
7AL-3338	675,025,714	R	B	B	R
	Homozygous recombinant	19.69	19.54	18.42	17.93
**Average**	Homo. WAPO1 B *	19.84	19.91	19.79	19.79
	Homo. WAPO1 R *	18.25	18.25	18.17	18.40
***P* values**	Homo. B	0.4325	0.0207	**1.2E-08**	**1.6E-06**
**homo-rec. *vs.***	Homo R	**3.4E-07**	**5.6E-09**	0.175	0.0463
	Infered SNS allele	B	B	R	R

* Average homozygous parent and all lines in trial carrying the corresponding allele (excluding family tested).

Figure S3

Haplotype analysis of the SNS QTL candidate region between flanking markers Treas1400-I4V (673,779,017) and IWA5913 (674,276,906). This is only part of the 2.3 Mb haplotype block. Exome capture data (deposited in T3 (https://triticeaetoolbox.org/wheat/) revealed 3 haplotypes.

Haplotype H1 included 13 common wheat lines and 4 tetraploid (4x) durum lines (1= 16REG01644, 2= Excalibur, 3= McNeal, 4= KS05HW14-3, 5= Reeder, 6= W7984, 7= Overley, 8= CO960293, 9= Vida, 10= RAC875, 11= C0940610, 12= Chateau, 13= MN99394-1, 14= D447-DW1 (4x), 15= 280-1-Yr15 (4x), 16= Kronos (4x), 17= Svevo (4x). **Haplotype H2** included 33 common wheat lines (1= Platte, 2= Berkut, 3= MN98550-5, 4= UI_Platinum, 5= RioBlanco, 6= Cheyenne, 7= PI610750, 8= Hank, 9= Lyman, 10= 16REG01643, 11= IDO444, 12= CCW3A37, 13= CCW3A49, 14= LCS_Star, 15= Cltr7635, 16= PBW343, 17= Kern, 18= Patwin-515HP, 19= Duster, 20= Bakahtawar94, 21= TAM111, 22= OPATA, 23= PI70613, 24= TAM112, 25= Billings, 26= RSI5, 27= SS-MPV57, 28= Inayama, 29= Altamo, 30= SY_Capstone, 31= Dharwar_Dry, 32= Dayn, 33= 2045A). **Haplotype H3** included three common wheat lines from the SE USA (1= 26R61, 2= LA95135, 3= AGS2000).

		H1																	H2																																		H3		
RefSeq v1.0	Gene name	1	2	3	4	5	6	7	8	9	10	11	12	13	14	15	16	17	1	2	3	4	5	6	7	8	9	10	11	12	13	14	15	16	17	18	19	20	21	22	23	24	25	26	27	28	29	30	31	32	33	1	2	3
673,779,017	TraesCS7A02G481400	G	G	G	G	G	G	G	G	G	G	G	G	G	G	G	G	G	A	A	A	A	A	A	A	A	A	A	A	A	A	A	A	A	A	A	A	A	A	A	A	A	A	A	A	A	A	A	A	A	A	G	G	G
673,779,128	TraesCS7A02G481400	C	C	C	C	C	C	C	C	C	C	C	C	C	C	C	C	C	T	T	T	T	T	T	T	T	T	T	T	T	T	T	T	T	T	T	T	T	T	T	T	T	T	T	T	T	T	T	T	T	T	C	C	C
673,779,143	TraesCS7A02G481400	T	T	T	T	T	T	T	T	T	T	T	T	T	T	T	T	T	C	C	C	C	C	C	C	C	C	C	C	C	C	C	C	C	C	C	C	C	C	C	C	C	C	C	C	C	C	C	C	C	C	T	T	T
673,779,248	TraesCS7A02G481400	T	T	T	T	T	T	T	T	T	T	T	T	T	T	T	T	T	C	T	T	T	T	C	T	T	C	C	T	C	C	T	T	T	T	T	T	T	T	T	T	T	T	T	T	T	T	T	T	T	T	T	T	T
673,779,333	TraesCS7A02G481400	T	T	T	T	T	T	T	T	T	T	T	T	T	T	T	T	t	A	A	A	A	A	A	A	A	A	A	A	A	A	A	A	A	A	A	A	A	A	A	A	A	A	A	A	A	A	A	A	A	A	T	T	T
673,779,339	TraesCS7A02G481400	T	T	T	T	T	A	T	T	T	T	T	T	T	A	A	A	-	T	T	T	T	T	T	T	T	T	T	T	T	T	T	T	T	T	T	T	T	T	T	T	T	T	T	T	T	T	T	T	T	T	T	T	T
673,779,357	TraesCS7A02G481400	A	A	A	A	A	A	A	A	A	A	A	A	A	a	a	a	a	T	T	T	T	T	T	T	T	T	T	T	T	T	T	T	T	T	T	T	T	T	T	T	T	T	T	T	T	T	T	T	T	T	T	T	T
673,993,810	TraesCS7A02G481400	G	G	G	G	G	G	G	G	G	G	G	G	G	G	G	g	G	G	G	G	G	G	G	G	G	G	G	G	G	G	G	G	G	G	G	G	G	G	G	G	G	G	G	G	G	G	G	g	g	G	A	A	a
673,993,852	TraesCS7A02G481400	A	A	A	A	a	A	A	A	A	A	A	A	A	A	A	a	A	G	G	G	G	g	G	G	G	G	G	G	G	G	G	G	G	G	G	G	G	G	G	G	G	G	G	G	G	G	G	g	G	G	A	a	a
674,015,961	TraesCS7A02G481500	C	C	C	C	C	C	C	C	C	C	C	C	C	C	C	C	C	T	T	T	T	T	T	T	T	T	T	T	T	T	T	T	T	T	T	T	T	T	T	T	T	T	T	T	T	T	T	T	T	T	T	T	T
674,016,168	TraesCS7A02G481500	T	T	T	T	T	T	T	T	T	T	T	T	T	T	T	T	T	C	C	C	C	C	C	C	C	C	C	C	C	C	C	C	C	C	C	C	C	C	C	C	C	C	C	C	C	C	C	C	C	C	C	C	C
674,018,102	TraesCS7A02G481500	C	C	C	C	C	C	C	C	C	C	C	C	C	C	C	C	C	C	C	C	C	C	C	C	C	C	C	C	C	C	C	C	C	C	C	C	C	C	C	C	C	C	C	C	C	C	C	C	C	C	G	G	G
674,018,335	TraesCS7A02G481500	C	C	C	C	C	C	C	C	C	C	C	C	C	C	C	C	C	C	C	C	C	C	C	C	C	C	C	C	C	C	C	C	C	C	C	C	C	C	C	C	C	C	C	C	C	C	C	C	C	C	T	T	T
674,018,426	TraesCS7A02G481500	C	C	C	C	C	C	C	C	C	C	C	C	C	C	C	C	C	C	C	C	C	C	C	C	C	C	C	C	C	C	C	C	C	C	C	C	C	C	C	C	C	C	C	C	C	C	C	C	C	C	G	G	G
674,018,824	TraesCS7A02G481500	C	C	C	C	C	C	C	C	C	C	C	C	C	C	C	C	C	C	C	C	C	C	C	C	C	C	C	C	C	C	C	C	C	C	C	C	C	C	C	C	C	C	C	C	C	C	C	C	C	C	A	A	A
674,019,484	TraesCS7A02G481500	T	T	T	T	T	T	T	T	T	T	T	T	T	T	T	T	T	T	T	T	T	T	T	T	T	T	T	T	T	T	T	T	T	T	T	T	T	T	T	T	T	t	T	T	T	T	T	T	T	T	C	C	c
674,019,603	TraesCS7A02G481500	G	G	G	G	G	G	G	G	G	G	G	G	G	G	G	G	G	G	A	G	A	A	G	G	A	G	G	G	G	G	A	G	-	A	A	G	G	G	G	G	G	G	G	G	G	-	G	G	G	G	G	G	G
674,019,951	TraesCS7A02G481500	G	g	G	G	g	G	G	G	G	G	G	G	G	g	G	g	g	a	A	A	A	A	A	A	A	A	A	A	A	A	A	A	A	A	A	A	A	A	A	A	A	A	A	A	A	A	A	A	A	A	G	G	G
674,020,022	TraesCS7A02G481500	C	c	C	C	C	C	C	C	C	C	C	C	C	c	c	c	c	T	T	T	T	T	T	T	T	T	T	T	T	T	T	T	T	T	T	T	T	T	T	T	T	T	T	T	T	T	T	T	T	T	T	T	T
674,082,248	TraesCS7A02G481600	C	C	C	C	C	C	C	C	C	C	C	C	C	C	C	C	C	C	C	C	c	C	C	C	C	C	C	C	c	C	C	C	C	C	C	C	C	c	C	C	C	C	C	C	C	C	C	C	C	C	T	t	T
674,090,707	TraesCS7A02G481700	A	A	a	A	A	A	A	A	A	A	A	A	a	A	A	A	A	T	T	T	T	T	T	T	T	t	T	T	T	T	T	T	T	T	T	T	T	T	T	T	T	T	T	T	T	T	T	T	T	T	A	a	A
674,090,816	TraesCS7A02G481700	T	T	T	T	T	T	T	T	T	T	T	T	T	T	T	T	T	T	T	T	T	T	T	T	T	T	T	T	T	T	T	T	T	T	T	T	T	T	T	T	T	T	T	T	T	T	T	T	T	T	C	C	C
674,091,310	TraesCS7A02G481700	G	G	G	G	G	G	G	G	G	G	G	G	G	G	G	G	G	G	G	G	G	G	G	G	G	G	G	G	G	G	G	G	G	G	G	G	G	G	G	G	G	G	G	G	G	G	G	G	G	G	A	A	A
674,091,312	TraesCS7A02G481700	T	T	T	T	T	T	T	T	T	T	T	T	T	T	T	T	T	T	T	T	T	T	T	T	T	T	T	T	T	T	T	T	T	T	T	T	T	T	T	T	T	T	T	T	T	T	T	T	T	T	A	A	A
674,091,635	TraesCS7A02G481700	C	C	C	C	C	C	C	C	C	C	C	C	c	C	C	C	C	T	T	T	T	T	T	T	T	T	T	T	T	T	T	T	T	T	T	T	T	T	T	T	T	T	T	T	T	T	T	T	T	T	T	T	T
674,091,656	TraesCS7A02G481700	T	T	T	T	T	T	T	T	T	T	T	T	T	T	T	T	T	A	A	A	A	A	A	A	A	A	A	A	A	A	A	A	A	A	A	A	A	A	A	A	A	A	A	A	A	A	A	A	A	A	T	T	T
674,091,822	TraesCS7A02G481700	C	C	C	C	c	C	C	C	C	C	C	C	C	C	C	C	c	C	C	C	C	C	C	C	C	C	C	C	c	C	C	C	C	C	C	C	C	C	C	C	C	C	C	C	C	C	C	C	C	C	T	T	T
674,106,327	TraesCS7A02G481800	G	G	G	G	G	G	G	G	G	G	G	G	G	G	G	G	G	A	A	A	A	A	A	A	A	A	A	A	A	A	A	A	A	A	A	A	A	A	A	A	A	A	A	A	A	A	A	A	A	A	A	A	A
674,106,471	TraesCS7A02G481800	t	T	T	T	t	T	T	T	T	T	T	T	T	T	T	T	T	G	G	G	G	G	G	G	G	G	G	G	G	G	G	G	G	G	G	G	G	g	G	g	G	G	G	G	G	G	G	G	G	G	G	G	G
674,109,269	TraesCS7A02G481800	C	c	c	C	C	C	C	c	C	c	C	C	C	c	c	c	c	T	T	T	T	T	T	T	T	t	T	t	T	T	T	T	T	T	T	T	T	T	T	T	T	T	T	T	T	t	T	T	T	T	T	T	T
674,109,327	TraesCS7A02G481800	G	g	G	G	G	G	G	G	G	g	G	G	G	G	g	g	g	A	A	A	A	A	A	A	A	A	A	A	A	A	A	A	A	A	A	A	A	A	A	A	A	A	A	A	A	A	A	A	A	A	A	A	A
674,114,953	TraesCS7A02G481900	G	G	G	G	G	G	G	G	G	G	G	G	G	G	G	G	G	A	A	A	A	A	A	A	A	A	A	A	A	A	A	A	A	A	a	A	A	a	A	A	A	A	A	A	a	A	A	A	A	A	a	A	A
674,139,261	TraesCS7A02G481900	C	C	C	C	C	C	C	C	C	C	C	C	C	C	C	C	C	T	T	T	T	T	T	T	T	T	T	T	T	T	T	T	T	T	T	T	T	T	T	T	T	T	T	T	T	T	T	T	T	T	C	C	C
674,139,408	TraesCS7A02G481900	G	G	G	G	G	G	G	G	G	G	G	G	G	G	G	G	G	A	A	A	A	A	A	A	A	A	A	A	A	A	A	A	A	A	A	A	A	A	A	A	A	A	A	A	A	A	A	A	A	A	A	A	A
674,139,446	TraesCS7A02G481900	A	A	A	A	A	A	A	A	A	A	A	A	A	A	A	A	A	A	A	A	A	A	A	A	A	A	A	A	A	A	A	A	A	A	A	A	A	A	A	A	A	A	A	A	A	A	A	A	A	A	G	G	G
674,139,479	TraesCS7A02G481900	A	A	A	A	A	A	A	A	A	A	A	A	A	A	A	A	A	G	G	G	G	G	G	G	G	G	G	G	G	G	G	G	G	G	G	G	G	G	G	G	G	G	G	G	G	G	G	G	G	G	G	G	G
674,139,580	TraesCS7A02G482000	C	C	C	C	C	C	C	C	C	C	C	C	C	C	C	C	C	T	T	T	T	T	T	T	T	T	T	T	T	T	T	T	T	T	T	T	T	T	T	T	T	T	T	T	T	T	T	T	T	T	T	T	T
674,139,626	TraesCS7A02G482000	G	G	G	G	G	G	G	G	G	G	G	G	G	G	G	G	G	A	A	A	A	A	A	A	A	A	A	A	A	A	A	A	A	A	A	A	A	A	A	A	A	A	A	A	A	A	A	A	A	A	G	G	G
674,140,270	TraesCS7A02G482000	G	G	g	G	G	G	g	G	G	G	G	G	G	G	G	G	G	A	A	A	A	A	A	A	A	A	A	A	A	A	A	A	A	A	A	A	A	A	A	A	A	A	A	A	A	A	A	A	A	A	G	G	G
674,268,110	TraesCS7A02G482000	T	T	T	T	T	T	T	T	T	t	T	T	t	T	T	t	T	T	T	T	T	t	T	T	T	T	T	T	T	T	T	T	T	T	T	T	T	T	T	T	T	T	T	T	T	T	T	T	T	t	A	a	A
674,268,162	TraesCS7A02G482000	G	G	G	G	G	G	G	G	g	g	G	G	g	G	G	g	G	A	A	A	A	a	A	A	A	A	A	A	A	A	A	A	A	A	A	A	A	A	A	A	A	A	A	A	a	A	A	A	a	a	A	A	A
674,268,165	TraesCS7A02G482000	G	G	G	G	G	G	G	G	g	g	G	G	g	G	G	g	G	G	G	G	G	g	G	G	G	G	G	G	G	G	G	G	G	G	G	G	G	G	G	G	G	G	G	G	g	G	G	G	g	g	T	T	T
674,270,369	TraesCS7A02G482000	G	G	G	G	G	G	G	G	G	G	G	G	G	G	G	G	G	G	G	G	G	G	G	G	G	G	G	G	G	G	G	G	-	G	G	G	G	G	G	G	G	G	T	G	G	G	T	T	T	G	G	G	G
674,272,225	TraesCS7A02G482000	T	T	T	T	T	T	T	T	T	t	T	T	T	T	T	T	T	G	G	G	G	G	G	G	G	G	G	G	G	G	G	G	G	G	G	G	G	G	G	G	G	G	G	G	G	G	G	G	G	G	G	G	G
674,273,096	TraesCS7A02G482000	G	G	G	G	G	G	G	G	G	G	G	G	G	G	G	g	G	C	C	C	C	C	C	C	C	C	C	C	C	C	C	C	C	C	C	C	C	C	C	C	C	C	C	C	C	C	C	C	C	C	C	C	C
674,274,216	TraesCS7A02G482000	C	C	C	C	C	C	C	C	C	C	C	C	C	C	C	C	C	G	G	G	G	G	G	G	G	G	G	G	G	G	G	G	G	G	G	G	G	G	G	G	G	G	G	G	G	G	G	G	G	G	G	G	G
674,274,219	TraesCS7A02G482100	G	G	G	G	G	G	G	G	G	G	G	G	G	G	G	G	G	A	A	A	A	A	A	A	A	A	A	A	A	A	A	A	A	A	A	A	A	A	A	A	A	A	A	A	A	A	A	A	A	A	G	G	G
674,274,282	TraesCS7A02G482100	G	G	G	G	G	G	G	G	G	G	G	G	G	G	G	G	G	A	A	A	A	A	A	A	A	A	A	A	A	A	A	A	A	A	A	A	A	A	A	A	A	A	A	A	A	A	A	A	A	A	G	G	G
674,274,822	TraesCS7A02G482100	C	C	C	C	C	C	C	C	C	C	C	C	C	C	C	C	C	C	C	C	C	C	C	C	C	C	C	C	C	C	C	C	C	C	C	C	C	C	C	C	C	C	C	C	C	C	C	C	C	C	T	T	T
674,275,218	TraesCS7A02G482100	C	C	C	C	c	C	C	C	C	c	C	C	C	C	C	c	c	T	T	T	T	T	T	T	T	T	T	t	T	T	T	T	t	t	t	T	t	T	t	T	T	T	T	T	T	T	T	T	T	t	C	C	C
674,275,457	TraesCS7A02G482100	T	T	t	T	T	T	T	T	t	T	T	t	T	T	T	t	T	C	C	C	C	C	C	C	C	C	C	C	C	C	c	C	C	C	C	C	C	c	C	C	C	C	C	C	C	C	c	C	C	C	C	C	C
674,275,459	TraesCS7A02G482100	C	C	c	C	C	C	C	C	C	C	C	c	C	C	C	c	C	C	C	C	C	C	C	C	C	C	C	C	C	C	c	C	C	C	C	C	C	c	C	C	C	C	C	C	C	C	c	C	C	C	T	T	T
674,275,583	TraesCS7A02G482100	C	C	c	c	c	C	C	C	c	C	C	c	C	C	C	C	C	C	C	T	C	C	C	C	C	C	C	C	C	C	C	C	C	C	C	C	C	C	-	C	C	C	T	C	C	T	T	T	T	C	C	C	C
674,275,611	TraesCS7A02G482100	G	G	g	g	g	G	G	G	g	G	G	g	G	G	G	G	G	A	A	A	A	a	A	A	A	A	A	A	A	A	A	A	A	A	A	A	A	A	a	A	A	A	A	A	A	A	A	A	A	A	G	G	G
674,275,613	TraesCS7A02G482100	C	C	c	c	c	C	C	C	c	C	C	c	C	C	C	C	C	A	A	A	A	a	A	A	A	A	A	A	A	A	A	A	A	A	A	A	A	A	a	A	A	A	A	A	A	A	A	A	A	A	C	C	C
674,276,906	TraesCS7A02G482100	G	G	G	G	g	G	G	G	G	g	G	G	G	G	G	G	G	a	A	A	A	A	A	A	A	A	A	A	A	A	A	A	A	a	A	A	A	A	A	A	A	A	A	a	A	A	A	A	A	A	G	G	G

Figure S4. Haplotype analysis of the photoperiod-insensitive spring wheat association mapping panel using the 90K SNP assay. SNPs within the 2.3 Mb haplotype block are indicated in gray.

SNP	RefSeq v1.0	Haplotype H1		Haplotype H2	
		HR07024_5	IDO629	H0900081	BERKUT
IWB6693	672,032,033	C	T	T	T
IWB29518	672,042,243	C	T	T	T
IWB7632	674,272,225	T	T	G	G
IWA5912	674,276,849	C	C	T	T
IWA5913	674,276,906	G	G	A	A
IWB54775	674,277,105	T	T	C	C
IWA7409	674,279,667	G	G	A	A
IWB5961	674,801,909	T	T	T	G
	N	41	6	134	74

Figure S5. WebLogo (https://weblogo.berkeley.edu/logo.cgi) from alignment of the closest WAPO1 F-BOX domain in 8 grass genera [a], and 46 non-grass genera [b]. At position 47 of WAPO1 (= position 15 of the WebLogo) all eleven grass genera have a cysteine (C47) and all non-grass genera have a phenylalanine (F47). This position is polymorphic in wheat between WAPO-A1b (F47) and all other alleles (C47).

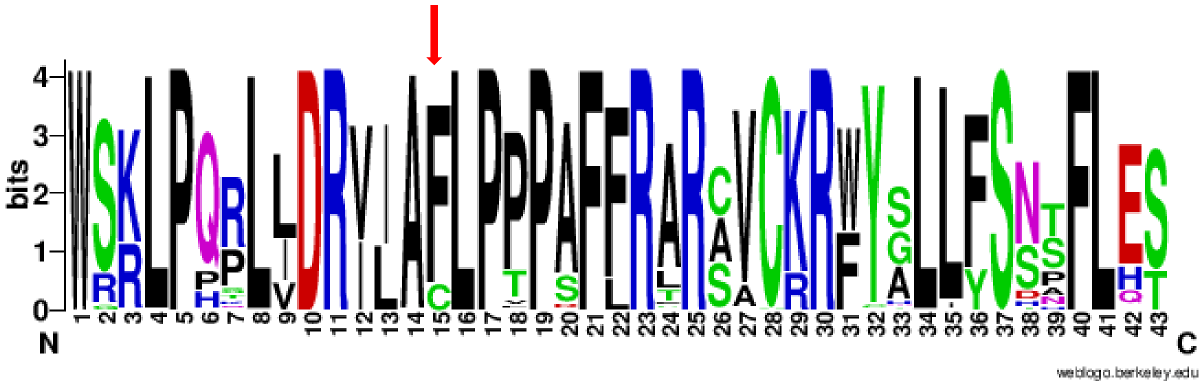

[a] 8 grass genera used: *Aegilops tauschii, Brachypodium distachyon, Dichanthelium oligosanthes, Oryza sativa, Panicum hallii, Setaria itálica, Sorghum bicolor, Zea mays.*

[b] 46 non-grass genera used: *Abrus precatorius, Ananas comosus, Arabidopsis thaliana, Arachis hypogaea, Artemisia annua, Beta vulgaris, Cajanus cajan, Camelina sativa, Capsella rubella, Capsicum annuum, Carica papaya, Chenopodium quinoa, Cicer arietinum, Citrus clementina, Coffea arabica, Corchorus capsularis, Cuscuta australis, Cynara cardunculus, Durio zibethinus, Eucalyptus grandis, Eutrema salsugineum, Glycine soja, Herrania umbrática, Hevea brasiliensis, Ipomoea nil, Juglans regia, Lactuca sativa, Lotus japonicus, Manihot esculenta, Medicago truncatula, Mucuna pruriens, Nelumbo nucifera, Nicotiana attenuata, Olea europea, Pisum sativum, Populus tomentosa, Quercus suber, Raphanus sativus, Ricinus communis, Spinacia oleracea, Theobroma cacao, Trifolium pratense, Vigna angularis, Vitis vinífera, Ziziphus jujube, Zostera marina.*

Supplementary Tables

Table S1. Description of spikelet number per spike (SNS) QTL identified in the Berkut x RAC875 RIL population. Values correspond to the combined analysis of four environments that were used as blocks. The four environments include Davis, CA and Imperial Valley, CA, under normal irrigation and terminal drought conditions. The percent of phenotypic variation explained (R^2) corresponds to a multiple QTL model as implemented in the R/qtl.

Trial	Chr. arm	Position cM	RefSeq v1.0	LOD	Additive effect [a]	R^2 (%)	*P* value
**Main effects**							
IWB61142	2BS	47.6	69,370,567	2.9	-0.81	13.0	2.42E-08
IWB9904	7AS	55.2	47,283,220	2.1	-0.69	9.1	4.16E-07
IWA7407	7AL	128.0	679,896,953	2.3	-0.75	10.1	3.34 E-07
**Interactions**							
7ALx7AS							0.62169
7ALx2BS							0.52512
7ASx2BL							0.33402

[a] A minus sign indicates that the Berkut allele increases spikelet number

Table S2. Sequenced accessions carrying the different *WAPO-A1* alleles (or with available genome sequences).

Allele	Comment	Accessions
WAPO-A1a	4x and 6x	Durum wheat: Ben and Kronos. Common wherat: RAC875, Clark, CO-940610, MN99394-1, Lancer, CDC Lanmark, Julius, Arina, Jagger, Cadenza, Robigus, and SY_Mattis.
WAPO-A1b	4x and 6x	Cultivated emmer: PI 41025, PI 480460 and PI 244341. Common wheat: Berkut, Ning7840, SS-MPV57, Platte, MN98550-5, CDC Stanley, Norin 61, Claire, and Mace.
WAPO-A1c	4x and 6x	Wild emmer: PI 471033 and PI 355455. Cultivated emmer: CItr 14135, PI 94638, and PI 298586. Durum wheat: PI 286539. Common wheat: AGS2000, LA95135 and 26R61.
WAPO-A1d	4x	Durum wheat: Rusty and Langdon. Cultivated emmer: CItr14919, PI 193877, PI 193882, PI 217640, PI 221400, PI 225332, PI 273980, and PI 94657.
